# Nitrogen Utilization and Ruminal Microbiota of Hu Lambs in Response to Varying Dietary Metabolizable Protein Levels

**DOI:** 10.3390/ani15142147

**Published:** 2025-07-21

**Authors:** Yitao Cai, Jifu Zou, Yibang Zhou, Jinyong Yang, Chong Wang, Huiling Mao

**Affiliations:** 1 College of Animal Science and Technology, Zhejiang A&F University, Hangzhou 311300, China; aa1430797479@163.com (Y.C.); 15968868271@163.com (J.Z.); 18357541389@163.com (Y.Z.); 2 College of Veterinary Medicine, Zhejiang A&F University, Hangzhou 311300, China; 3 Jixian Honors College, Zhejiang A&F University, Hangzhou 311300, China; 4 Zhejiang Provincial Animal Husbandry Technology Promotion and Monitoring Station of Breeding Livestock and Poultry, Hangzhou 310021, China; vineus425@gmail.com

**Keywords:** amino acid balance, metabolizable protein optimization, growth performance, nitrogen retention efficiency, rumen bacteria

## Abstract

Optimizing the metabolizable protein level in ruminant diets represents a promising strategy to increase nitrogen use efficiency and mitigate environmental pollution. This study explored the impacts of varying metabolizable protein (MP) levels on amino acid (AA) balance, nitrogen (N) utilization, and the ruminal microbiota in Hu lambs. Fifty-four female Hu lambs of 60 d old, with an average body weight (BW) of 18.7 ± 2.37 kg, were randomly allocated to three dietary MP groups: (1) low MP (LMP, 7.38% of DM), (2) moderate MP (MMP, 8.66% of DM), and (3) high MP (HMP, 9.93% of DM). Three lambs with similar BW within each group were housed together in a single pen, serving as one experimental replicate (*n* = 6). The feeding trial lasted for 60 days with 10 days for adaptation. The final BW of lambs in the MMP and HMP groups increased (*p* < 0.05) by 5.64% and 5.26%, respectively, compared to the LMP group. Additionally, lambs fed the MMP diet exhibited an 11.6% higher (*p* < 0.05) average daily gain than those in the LMP group. Increasing dietary MP levels enhanced (*p* < 0.05) N intake, urinary N, retained N, and percent N retained, but decreased apparent N digestibility (*p* < 0.05). Urinary uric acid, total purine derivatives, intestinally absorbable dietary protein, microbial crude protein, intestinally absorbable microbial crude protein, and actual MP supply all increased (*p* < 0.05) with higher MP values in the diet. The plasma concentrations of arginine, lysine, methionine, phenylalanine, threonine, aspartic acid, proline, total essential AAs, and total nonessential AAs were the lowest (*p* < 0.05) in the LMP group. In the rumen, elevated MP levels led to a significant increase (*p* < 0.05) in the ammonia N content. The relative abundances of *Candidatus_Saccharimonas*, *Ruminococcus*, and *Oscillospira* were the lowest (*p* < 0.05), whereas the relative abundances of *Terrisporobacter* and the *Christensenellaceae_R-7_group* were the highest (*p* < 0.05) in the MMP group. In conclusion, the moderate dietary metabolizable protein level could enhance growth performance, balance the plasma amino acid profiles, and increase nitrogen utilization efficiency in Hu lambs, while also altering the rumen bacterial community by increasing beneficial probiotics like the *Christensenellaceae_R-7_group*.

## Simple Summary

Metabolizable protein may be a good indicator of protein utilization by ruminants. In this study, Hu lambs were utilized to evaluate the effects of varying levels of metabolizable protein on nitrogen utilization, amino acid balance, rumen fermentation, and ruminal microbiota. The results indicated that optimizing the dietary metabolizable protein level significantly improved growth performance, balanced plasma amino acid profiles, and increased nitrogen utilization efficiency. The rumen bacterial community and its structure were also altered, with a notable increase in the *Christensenellaceae_R-7_group* abundance in the moderate metabolizable protein group, a microbial taxon recognized for its probiotic role in the rumen ecosystem. Based on comprehensive evaluation of physiological responses, microbial dynamics, and environmental impact, we recommend 8.66% dietary MP as the optimal level for 2–4-month-old female Hu lambs.

## 1. Introduction

In ruminant diets, protein plays a crucial role, not only in reducing the cost of production but also in influencing nitrogen (N) emissions to the environment. Owing to the activity of rumen microbes, protein metabolism in ruminants differs from monogastrics in that proteins pass through the rumen where they may be degraded and resynthesized with varying efficiency [1]. Based on their susceptibility to ruminal breakdown, dietary proteins are classified into rumen degradable protein (RDP) and rumen undegradable protein (RUP) [2]. RDP is enzymatically degraded by microbes into peptides, amino acids, and ammonia (NH_3_). The NH_3_ is then converted into microbial protein (MCP), which, together with RUP and a small fraction of endogenous protein, progresses to the intestinal tract for enzymatic digestion and absorption as amino acids and peptides [3]. In this case, metabolizable protein (MP), consisting of RUP, MCP, and minimal endogenous protein [2], more accurately reflects true protein utilization in ruminants compared to crude protein content alone. Notably, MCP supplies 50–80% of the absorbable true protein available to the host animal [4]. Feeding high-protein diets is a common practice to maximize animal productivity, while overfeeding protein can reduce N efficiency and increase N excretion [5]. In a study on cows, feed efficiency increased, but N use efficiency (g milk N/100 g N intake) demonstrated a linear decline (up to 5.4 percentage units), whereas urinary N excretion (g/100 g N intake) increased linearly with progressively higher MP supply levels (85%, 100%, and 115% of requirement) [6]. Importantly, research has demonstrated that optimizing dietary MP levels can improve N utilization and reduce nitrogen excretion and consequent environmental pollution [7].

On the other hand, ruminants maintain diverse and complex ruminal microbiota, which play crucial roles in converting fiber-rich plants and nonprotein nitrogen into short-chain fatty acids and microbial proteins [8,9]. A complex interrelationship exists among dietary nutrients, the rumen microbial composition, fermentation function, and the physiological activities of ruminants [10]. For example, the ruminal microbiota in weaned lambs are sensitive to dietary protein. *Prevotella*, *RC9_gut_group*, *Succinivibrionaceae_uncultured*, and *Veillonellaceae_uncultured* were the predominant bacterial genera in the rumen of lambs fed a high-protein diet (15% of DM), whereas *S24-7_norank* and *Ruminococcus* showed significantly higher relative abundance in lambs receiving a low-protein diet (11.7% of DM) [10].

Therefore, three experimental diets with varying MP levels (low MP [LMP], moderate MP [MMP], and high MP [HMP]) in our study were conducted in Hu lambs, a locally protected Chinese breed, to investigate their effects on N utilization efficiency, plasma amino acid (AA) profiles, rumen fermentation characteristics, and ruminal microbiota composition. Especially, our study offers a detailed analysis of how dietary MP influences the composition, diversity, and functional dynamics of the ruminal microbiota in lambs. We also hypothesized that (1) optimizing dietary MP levels may enhance N utilization efficiency and minimize N excretion to the environment and that (2) increasing dietary MP levels would induce changes in the composition of the ruminal microbiota.

## 2. Materials and Methods

### 2.1. Animals, Feeds, and Experimental Design

This study included fifty-four healthy female Hu lambs (mean body weight [BW] = 18.7 ± 2.37 kg at 60 days of age), which were randomly divided into three experimental groups with different MP levels: (1) LMP (7.38% of DM), (2) MMP (8.66% of DM), and (3) HMP (9.93% of DM), with 18 lambs per group. Three lambs with similar BW within each group were housed together in a single pen (2.5 m × 1.8 m), constituting one experimental replicate (*n* = 6). The feeding trial was conducted over a 60-day period, including a 10-day adaptation phase. In all the groups, feeds (Table 1) were given twice a day at 0800 and 1600. The lambs had free access to diet and water. The amounts of feed offered and refused were measured for two consecutive days biweekly, and the BWs of animals were also weighed every two weeks before the morning feeding. Daily rations were adjusted each morning to maintain refusal rates at 5–10% of the offered amount.

### 2.2. Nitrogen Metabolism

On the 25th day of the experiment, a digestion and metabolism test was carried out on all the lambs in their original pen for 5 days. The feed offered was adjusted to ensure approximately 5% feed refusal. Feces were collected using a plastic mesh basket placed at the bottom of pens, while urine was collected through a funnel into a glass bucket containing 10% (vol/vol) H_2_SO_4_ to maintain the final pH below three. The feces and urine were weighed daily in the morning before feeding. Approximately 5% of the daily feces, urine, and diet of each pen were collected and stored at −20 °C for subsequent analysis.

### 2.3. Chemical Composition

The feed and fecal samples were oven-dried at 60 °C for 72 h and stored in plastic containers at 4 °C until analysis. All samples were homogenized by grinding through a 1 mm mesh sieve using a Wiley mill (Arthur H. Thomas Co., Philadelphia, PA, USA) before analysis for dry matter (method 934.01) [11], N (method 954.01) [11], ash (method 942.05) [11], neutral detergent fiber [12], acid detergent fiber (method 973.18) [11], and ether extract (method 920.39) [11]. The N concentration in acidified urine samples was measured using micro-Kjeldahl analysis [11], while urinary purine derivatives (allantoin, uric acid, xanthine, and hypoxanthine) were analyzed using the George et al. [13] method.

### 2.4. Estimation of the Microbial Crude Protein Yield and MP

The microbial crude protein (MCP) was indirectly estimated via the urinary purine derivative (PD) [14]. The actual MP was calculated via the following equation: actual MP = intestinally absorbable rumen microbial protein (IAMCP) + intestinally absorbable dietary protein (IADP). IAMCP was computed using the formula IAMCP = MCP × 0.8 [15], whereas IADP was estimated via the equation IADP = RUP × intestinal digestibility of RUP (IDP). The IDP was determined through Gargallo et al. [16]’s modified three-step approach.

### 2.5. Blood Sample Collection and Analysis

At the end of the feeding trial, blood samples were collected from randomly selected lambs (one per replicate) in each of the three treatment groups prior to morning feeding (n = 6). Using heparinized tubes, 10 mL blood samples were obtained from the jugular vein of each animal. The samples were then centrifuged at 3000× *g* at 4 °C for 15 min to obtain the plasma. The plasma samples were analyzed for plasma amino acid (AA) concentrations via a Hitachi L-8900 amino acid analyzer (Hitachi, Tokyo, Japan).

### 2.6. Rumen Fermentation Parameters Analysis

On day 50, the lambs selected for blood sample collection were also used to collect rumen fluid samples (n = 6). Approximately 30 mL of rumen fluid was collected via rumen intubation prior to morning feeding, with the initial 15 mL discarded to prevent potential saliva contamination [17]. The remaining rumen fluid was filtered through four layers of sterile gauze. Approximately 20 mL of the strained fluid was stored at −20 °C for rumen fermentation parameters analysis, while the other 10 mL was preserved at −80 °C for analysis of ruminal microbiota.

The concentrations of volatile fatty acids (VFAs) were measured using an Agilent 7890A gas chromatography system (Agilent Technologies, Palo Alto, CA, USA) according to the method described by Hu et al. [18]. Ruminal ammonia-N (NH_3_-N) concentration was measured through steam distillation into boric acid followed by titration with dilute hydrochloric acid.

### 2.7. Analysis of Ruminal Microbiota

The total genomic DNA from the rumen fluid samples was extracted using the E.Z.N.A.^®^ Soil DNA Kit (Omega Bio-Tek, Norcross, GA, USA). The hypervariable V3-V4 region of the bacterial 16S rRNA gene was amplified with the universal primers 338F (5′-ACTCCTACGGGAGGCAGCA-3′) and 806R (5′-GGACTACHVGGGTWTCTAAT-3′). The resulting PCR products were quantified with the Quant-iT PicoGreen dsDNA Assay Kit (Invitrogen, Carlsbad, CA, USA). Subsequently, the amplicon libraries were generated by pooling equimolar amounts of PCR products and were subjected to paired-end sequencing (2 × 300 bp) on an Illumina NovaSeq platform at Novogene Bioinformatics Technology Co., Ltd. (Tianjin, China).

After assembly of paired-end reads with FLASH, sequences were demultiplexed and assigned to each sample using the individual unique barcode via Quantitative Insight into Microbial Ecology (QIIME). OTU clustering was performed at 97% similarity, with taxonomic assignment carried out against the SILVA138 SSU rRNA database. Alpha diversity measurements, including the Chao1, Shannon, Simpson, and PD_whole_tree indices, were implemented in QIIME. The shared and unique species across groups were visualized using a Venn diagram. To assess microbial community dissimilarity influenced by varying MP levels, principal coordinate analysis (PCoA) was conducted based on weighted UniFrac distances. Significant differences in bacterial taxa among the three groups were identified through linear discriminant analysis effect size (LEfSe). Additionally, the functional potential of the rumen microbiota was predicted by using Phylogenetic Investigation of Communities by Reconstruction of Unobserved States (PICRUSt). Briefly, following normalization of each OTU’s abundance based on marker gene copy numbers, functional predictions were performed using the Kyoto Encyclopedia of Genes and Genomes (KEGG) database [19].

### 2.8. Statistical Analysis

A completely randomized design was employed for data analysis, with each pen (containing three lambs) representing one experimental unit for the analysis of BW, dry matter intake (DMI), and N metabolism data, whereas each randomly chosen lamb from individual pens constituted an independent experimental unit for the analysis of plasma AA, rumen fermentation parameters, and the ruminal bacterial community. The growth performance parameters, plasma AA concentrations, and rumen fermentation characteristics were analyzed using one-way ANOVA in SPSS statistic software (version 26.0; SPSS Inc., Chicago, IL, USA). Duncan’s comparison was used to assess differences among treatment groups. For microbial community analysis, the alpha-diversity indices and bacterial relative abundance (phylum and genus levels) were compared using the Kruskal–Wallis rank-sum test. Between groups, differences were evaluated using analysis of similarities, with statistical interpretation as follows: significant difference was defined as R > 0.5 with *p* < 0.05, while 0.3 < R < 0.5 with *p* < 0.05 was considered indicative of a trend, and no significant difference was considered when R < 0.3. A linear discriminant analysis (LDA) score greater than 3, as calculated by LEfSe, was considered to show an important contributor. The correlations between differentially abundant bacterial genera and phenotypic variables were compared, and a correlation heat map was generated using ChiPlot (https://www.chiplot.online/mantel_test_correlation_heatmap.html) (accessed on 2 April 2025). Statistical significance was set at *p* < 0.05, while results with 0.05 ≤ *p* < 0.10 were considered statistical trends.

## 3. Results

### 3.1. Growth Performance

No significant differences were detected in initial BW, DMI, or feed efficiency among the three groups (*p* > 0.05, Table 2). However, the final BW of lambs in the MMP and HMP groups increased (*p* < 0.05) by 5.64% and 5.26%, respectively, compared to the LMP group. Additionally, the MMP-fed lambs showed significantly higher (11.6%, *p* < 0.05) average daily gain (ADG) than those fed the LMP group.

### 3.2. Nitrogen Utilization

As expected, N intake, urinary N, retained N, percent N retained, and N retained per 100 g body weight gain increased (*p* < 0.01) with increasing dietary MP supply (Table 3). However, as dietary MP level increased, apparent N digestibility significantly decreased (*p* < 0.05) by 17.2% and 29.0% in the MMP and HMP groups, respectively. Fecal N levels did not differ significantly among the groups (*p* > 0.05).

The HMP group exhibited significantly higher levels of uric acid (+0.43 mg/d), xanthine and hypoxanthine (+1.24 mg/d), total PD (+2.48 mg/d), MCP (+14 g/d), IAMCP (+11.2 g/d), and IADP (+13.4 g/d) compared to the LMP group (Table 4). However, no statistically significant differences (*p* > 0.05) were observed in these parameters between the LMP and MMP groups. The actual MP in the MMP and HMP groups increased (*p* < 0.05) by 14% and 37.8%, respectively, compared to the LMP group.

### 3.3. Plasma Free AA

The plasma concentrations of total essential AAs, total nonessential AAs, and most AAs including arginine (Arg), lysine (Lys), methionine (Met), phenylalanine (Phe), threonine (Thr), aspartic acid (Asp), and proline (Pro) were significantly greater (Table 5; *p* < 0.05) in the MMP and HMP groups than those in the LMP group. However, no significant differences (*p* > 0.05) in these AAs were detected between the MMP and HMP groups. In contrast, the LMP group exhibited 7.00% and 4.48% greater plasma cysteine concentrations (*p* < 0.05) than the MMP and HMP groups, respectively.

### 3.4. Rumen Fermentation Parameters

In the rumen, no significant differences in the concentrations of total VFAs, acetate, propionate, butyrate, iso-valerate, or valerate were detected among the groups (*p* > 0.05; Table 6). However, the HMP group showed significantly higher iso-butyrate levels than the LMP and MMP groups (*p* < 0.01). Additionally, the NH_3_-N content significantly increased (from 129 mg/L to 170 mg/L) with increasing MP levels in the diet (*p* < 0.001).

### 3.5. Bacterial Community Composition of the Rumen Microbiota

**Figure 1:**
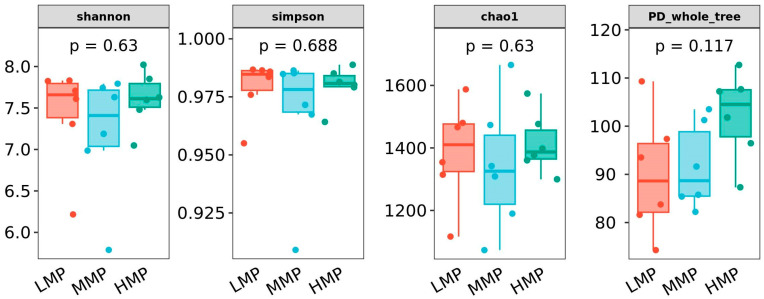
Ruminal microbiota alpha diversity of Hu lambs. LMP = low metabolizable protein; MMP = moderate metabolizable protein; HMP = high metabolizable protein.

A total of 1,686,140 raw reads were obtained from lamb rumen fluid samples. Following quality control processing, 1,074,629 high-quality effective reads were retained, with an average of 63,213 ± 5081 reads per sample. The rarefaction curves tended toward saturation (Figure S1A), indicating that the sequencing depth adequately captured the microbial diversity. On the basis of 97% similarity, 3805 operational taxonomic units (OTUs) were annotated, including 1771 bacterial OTUs shared across all the samples (Figure S1B). No significant differences in alpha diversity indices were detected among the three groups (*p* > 0.05, Figure 1).

**Figure 2:**
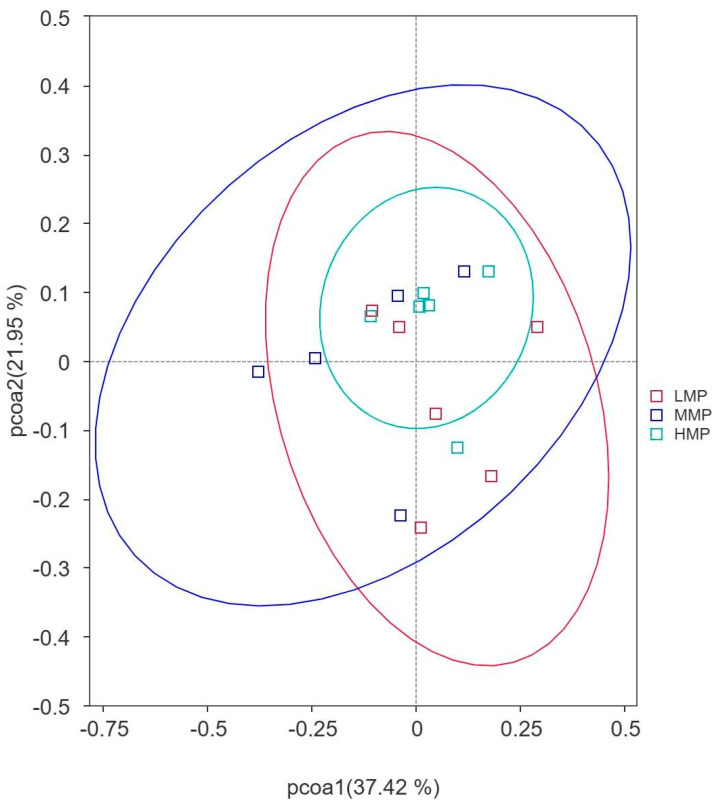
Principal coordinate analysis (PCoA) based on Weighted UniFrac distance. LMP = low metabolizable protein; MMP = moderate metabolizable protein; HMP = high metabolizable protein.

**Figure 3:**
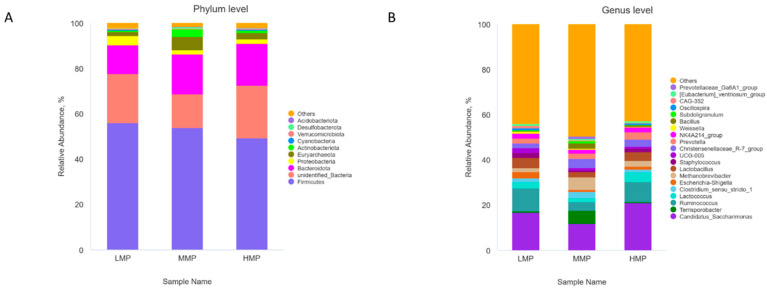
Effects of dietary metabolizable protein levels on phylum-level (**A**) and genus-level (**B**) composition of the rumen microbiota. The top 10 phyla and the top 20 genera are listed. LMP = low metabolizable protein; MMP = moderate metabolizable protein; HMP = high metabolizable protein.

**Figure 4:**
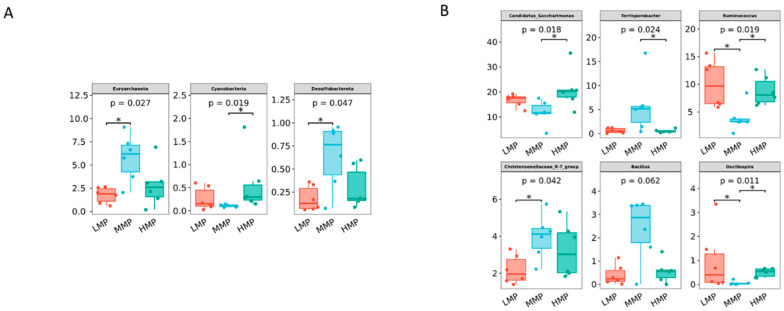
Comparisons of relative abundances at the phylum (**A**) and genus (**B**) levels. They were analyzed by the Kruskal–Wallis rank-sum test. * *p* < 0.05. LMP = low metabolizable protein; MMP = moderate metabolizable protein; HMP = high metabolizable protein.

**Figure 5:**
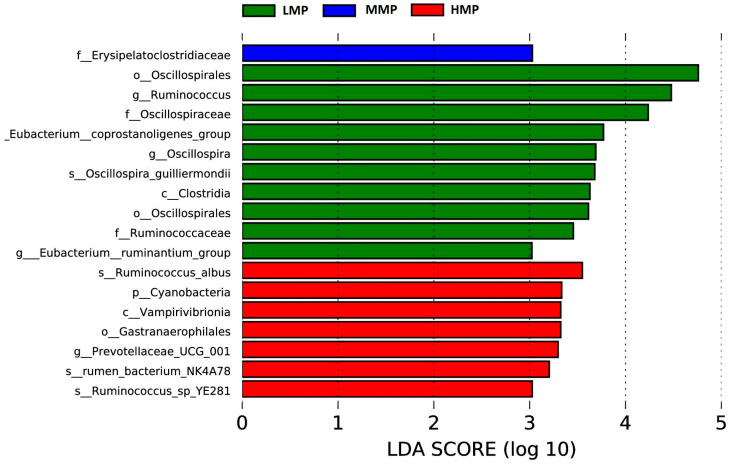
The ruminal microbial community composition was modulated by different dietary metabolizable protein levels and key phylotypes of the ruminal microbiota among the three groups. Linear discriminant analysis (LDA) score: taxa with LDA scores greater than 3 are shown in the histogram; the greater the LDA score was, the more significant the phylotype microbiota was in the comparison. LMP = low metabolizable protein; MMP = moderate metabolizable protein; HMP = high metabolizable protein.

Principal component analysis showed that PCo1 (37.42%) and PCo2 (21.95%) explained the observed variation among the samples (Figure 2). The rumen bacterial community in the MMP group tended to differ from that in the HMP group (R = 0.333, *p* = 0.013). However, there were no significant differences between the LMP and MMP groups (R = 0.269, *p* = 0.024) or between the LMP and HMP groups (R = 0.061, *p* = 0.247). At the phylum level, Firmicutes, unidentified_bacteria, and Bacteroidetes were the dominant phyla across all groups (Figure 3A). The relative abundances of Euryarchaeota and Desulfobacterota were the highest (*p* < 0.05), whereas Cyanobacteria was the lowest (*p* < 0.05) in the MMP group (Figure 4A). The 20 most abundant genera in the rumen of the lambs are presented in Figure 3B. The relative abundances of *Candidatus_Saccharimonas*, *Ruminococcus*, and *Oscillospira* were the lowest (*p* < 0.05), whereas the relative abundances of *Terrisporobacter* and the *Christensenellaceae_R-7_group* were the highest (*p* < 0.05) in the MMP group (Figure 4B). To determine the key phylotypes of ruminal microbiota among the three groups, we conducted LEfSe analysis (Figure 5). The LDA score results demonstrated ten discriminative features in the LMP group, with *Oscillospirales*, *Ruminococcus*, and *Oscillospiraceae* being the predominant microbiota. The MMP group was characterized by one dominant microorganism, *Erysipelatoclostridiaceae*. The HMP group exhibited seven discriminative features, with *Ruminococcus_albus* and *Cyanobateria* as the major microbiota.

### 3.6. Predicted Rumen Microbial Functions

**Figure 6:**
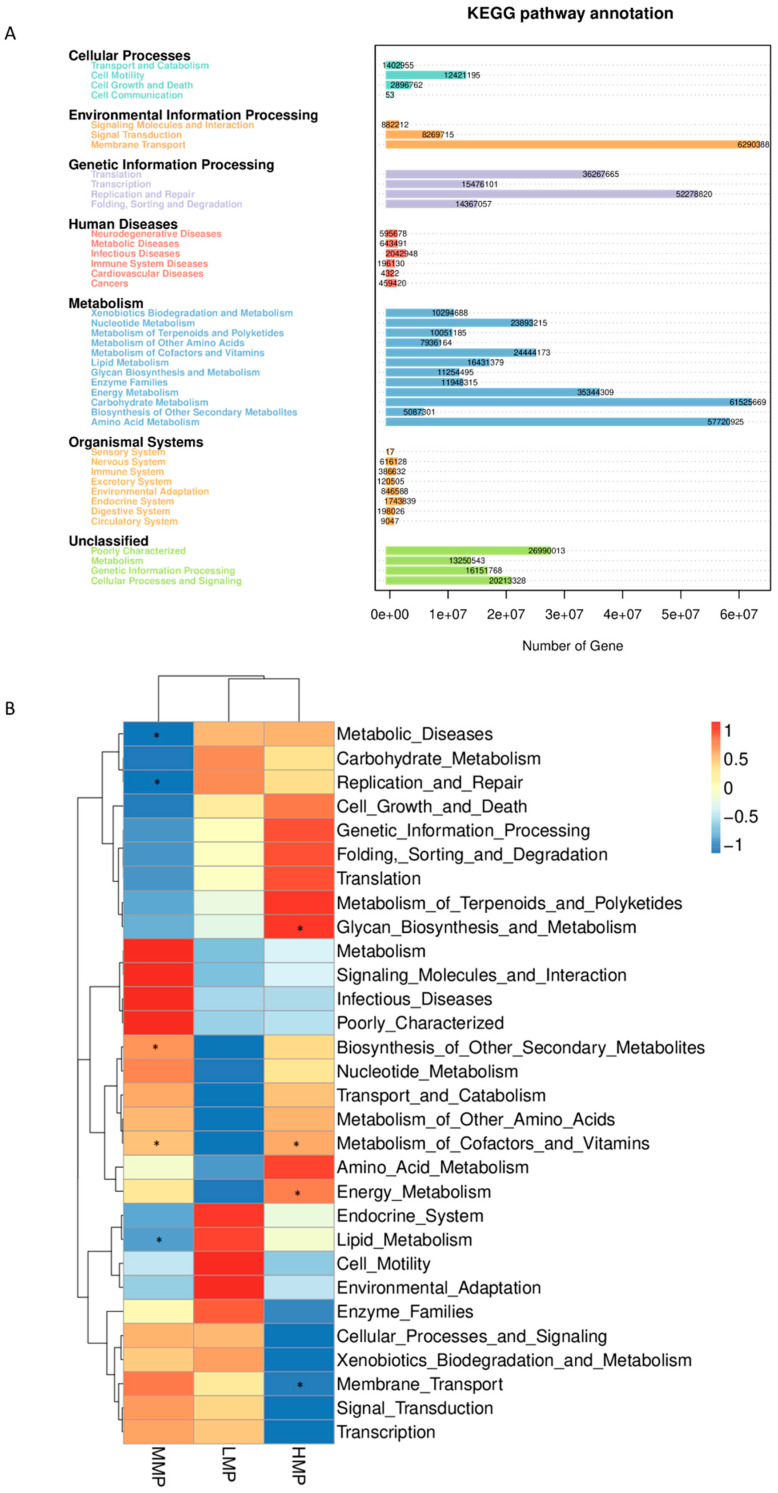
Predicted functional features (based on Kyoto Encyclopedia of Genes and Genomes orthologs and the Greengenes database 13_8). (**A**) Abundances of the differential metabolic pathways. (**B**) Changes in the predicted metagenomic functions of rumen bacteria in lambs with different dietary metabolizable protein levels. * represents a significant difference (*p* < 0.05). All comparisons were made with the LMP group. Red represents increased and blue represents decreased. LMP = low metabolizable protein; MMP = moderate metabolizable protein; HMP = high metabolizable protein.

To assess the impact of different dietary MP levels on the functional variations of ruminal microbiota in Hu lambs, we performed a functional analysis of the microbiota via Phylogenetic Investigation of Communities by PICRUSt. The abundance statistics of functions revealed that the majority of microbial functions were associated with metabolism (Figure 6A). The top six predicted functions for the rumen microbiota across all groups were “membrane transport” (11.1%), “carbohydrate metabolism” (10.8%), “amino acid metabolism” (10.2%), “replication and repair” (9.2%), “translation” (6.38%), and “energy metabolism” (6.23%). In the MMP group, the functions “biosynthesis of other secondary metabolites” and “metabolism of cofactors and vitamins” were enriched (*p* < 0.05; Figure 6B), whereas “metabolic diseases”, “replication and repair”, and “lipid metabolism” were decreased (*p* < 0.05) compared with those in the LMP group. In the HMP group, the functions “glycan biosynthesis and metabolism”, “metabolism of cofactors and vitamins”, and “energy metabolism” were enhanced (*p* < 0.05), whereas “membrane transport” was decreased (*p* < 0.05) relative to those in the LMP group.

### 3.7. Relationships Between Bacterial Taxa and Phenotypic Variables

**Figure 7:**
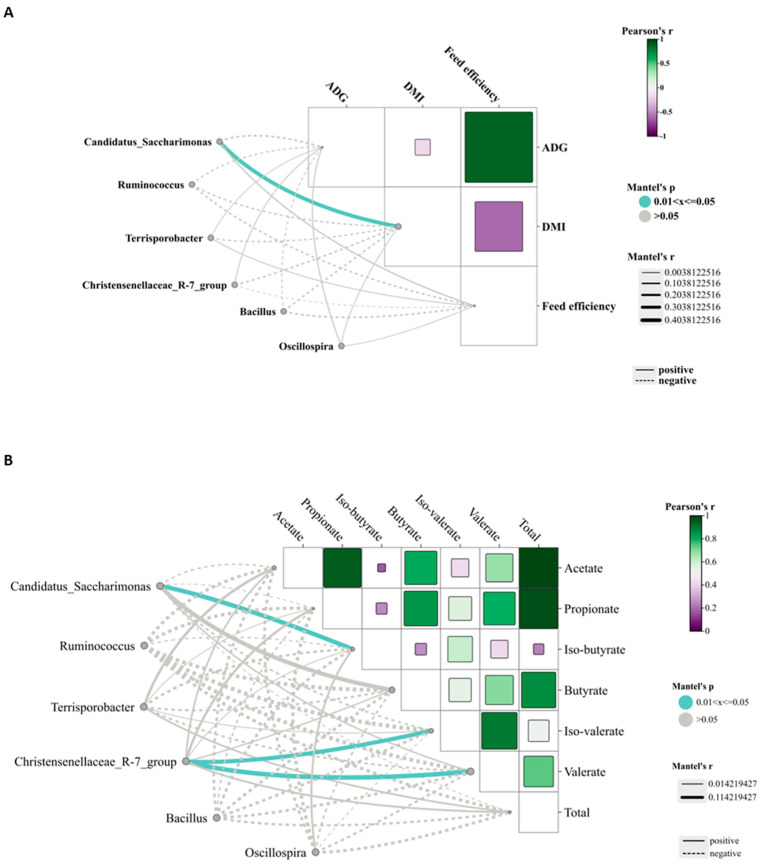
Correlation analysis of differentially abundant bacterial genera with phenotypic variables. (**A**) Correlation analysis of differentially abundant bacterial genera with growth performance. (**B**) Correlation analysis of differentially bacterial genera with ruminal volatile fatty acids.

Correlation analysis revealed a significant positive association (*p* < 0.05) between DMI and the relative abundance of *Candidatus_Saccharimonas* (Figure 7A). Furthermore, the relative abundance of *Candidatus_Saccharimonas* was significantly positively correlated (*p* < 0.05) with the ruminal iso-butyrate concentration (Figure 7B). Additionally, significant positive correlations (*p* < 0.05) were observed between the concentrations of iso-valerate and valerate and the abundance of the *Christensenellaceae_R-7_group* (Figure 7B).

## 4. Discussion

Nitrogen is a vital nutrient that plays a critical role in the growth and productivity of ruminants, but its inefficient utilization leads to excessive excretion and environmental pollution [9]. The use of the MP system, rather than the CP system, aligns more closely with the actual protein needs of Hu lambs and plays an important role in reducing N emission, thereby promoting environmental sustainability in intensive livestock production. The protein level in feed should match the physiological demands of the animal to ensure adequate protein utilization and absorption [20]. The growth rate and energy efficiency can be improved by providing sufficient MP to meet the requirements for genetic growth potential under optimal energy intake [21]. In the current study, the ADG of Hu lambs increased with increasing dietary MP levels, aligning with previous research findings that ADG is closely associated with dietary N, rumen fermentable carbohydrates, and the intestinal MP supply [22]. Nevertheless, no change in DMI was observed despite the increase in MP supply, a finding that is consistent with studies on dairy cows [23,24]. When dietary MP levels align with the metabolic demands of Hu lambs, N retention improves, reducing waste excretion. This could explain why higher MP diets in our study enhanced ADG without increasing DMI, a potential metabolic adaptation where improved protein utilization optimized growth efficiency rather than stimulating feed consumption.

Previous studies have demonstrated that high-protein diets significantly increase urinary N excretion while exerting limited effects on fecal or milk N outputs [25,26]. As urinary N constitutes the predominant route of N excretion, it serves as a robust biomarker for assessing animal N utilization efficiency. Urinary urea-N is rapidly converted into ammonium and volatilized as ammonia, contributing to environmental pollution [27]. Consequently, optimizing dietary MP levels is crucial for mitigating environmental issues while maintaining animal productivity. Elevated dietary CP levels proportionally increase N intake in ruminants [28,29]; this positive correlation between dietary MP levels and N retention was similarly demonstrated in our current study. However, while CP reduction may decrease N excretion, it concurrently imposes growth performance limitations [30]. Notably, a previous study has documented that apparent N digestibility is greater in animals under low dietary protein [31], a finding corroborated by Ouellet and Chiquette’s report [32] of improved N digestibility despite reduced MP intake in dairy cattle. Furthermore, the N retention rate from high dietary protein intake was higher [28,33].

The excretion of PD exhibited significant dependence on dietary CP levels [14]. Rumen microorganisms utilize N and carbon sources to synthesize MCP, with the MCP quantity increasing as PD levels rise [34]. Both PD excretion and MCP synthesis were shown to be positively correlated with increasing dietary CP content [35], as elevated protein provision enhances N substrate availability for microbial proliferation, consequently increasing both RDP and RUP quantities. Notably, our results revealed that the actual MP synthesized by female Hu lambs was lower than that predicted by the NRC standards [15], likely due to the young age of the animals used in this study and their underdeveloped rumen, as well as a lower efficiency of dietary CP utilization by rumen microorganisms than that in adult sheep.

The MP requirements are fundamentally determined by the efficiency of various physiological processes, including maintenance, lactation, growth, and pregnancy [36,37]. Lee et al. [38] reported that decreasing MP supply while maintaining balanced AA profiles in dairy diets decreased the urinary N and urea-N excretion, suggesting that the AA balance might be more important than the MP supply. Modifying dairy cow diets to lower total protein content while maintaining adequate AA balance can effectively mitigate N excretion’s environmental impact, lower feed costs, and sustain milk production [39]. Räisänen et al. [40] found that when dairy cows were fed an MP-adequate diet, the supply of dHis did not influence milk true protein concentration or yield, while milk fat and energy-corrected milk yields were optimized at dHis supply of 69 g/d or 2.65% of MP. These findings prompted our investigation of plasma AA profiles to assess AA utilization efficiency. AA requirements are primarily governed by two key factors: protein retention capacity and nitrogen excretion patterns [41]. Our results revealed that dietary MP deficiency in the LMP group significantly reduced plasma concentrations of several critical AAs, including Arg, Lys, Met, Phe, Thr, Asp, and Pro, as well as total essential AAs and non-essential AAs compared to the MMP and HMP groups. Lys, Met, and Thr, as limiting AAs, are critical for the growth, physiology, and reproductive performance of calves [42]. The current findings demonstrated a positive association between growth performance and plasma concentrations of these limiting AAs, with the MMP and HMP groups showing higher levels than the LMP group. Lambs fed the LMP diet had the lowest plasma Pro concentration. This might be related to the lowest Lys digestible flow with the MP diets, as Lys serves as an important precursor for de novo Pro synthesis [43]. Interestingly, the plasma AA profiles observed between the MMP and HMP groups suggested that AA absorption and utilization reached saturation at MMP levels, indicating that the dietary MP provision in the MMP group likely represents the optimal threshold for these growing lambs.

Dietary MP levels did not alter rumen fermentation patterns, as indicated by stable concentrations of total VFAs and individual VFAs, with the exception of iso-butyrate. Iso-butyrate, a degradation product of feed protein in the rumen, increases with increasing MCP synthesis [44]. He et al. [45] demonstrated that high-CP diets increased the abundance of iso-butyrate-producing bacteria, consequently promoting iso-butyrate production. Furthermore, supplemental levels of iso-butyric acid were found to increase the effective degradation rates of cellulose and hemicellulose while decreasing the effective degradation rate of crude protein [46]. This may account for the observed reduction in apparent N digestibility with increasing dietary MP levels in the present study. Ruminal ammonia generally reflects the extent of protein degradation. More protein of the same degradability means more rumen ammonia [47]. Previous studies have established that elevated dietary crude protein levels lead to increased ruminal NH_3_-N concentrations, confirming a direct positive relationship between protein intake and NH_3_-N production [48]. Interestingly, Benchaar et al. [6] reported that ruminal NH_3_ concentration and urinary purine derivatives remained unchanged across different MP supply levels, indicating stable microbial protein synthesis. This stability likely resulted from sufficient RDP provision in their study. Importantly, our findings suggest that the improved animal performance observed with higher MP supply primarily stems from increased contributions of RUP to the metabolizable protein pool.

*Candidatus_Saccharimonas* has emerged as the dominant genus and shows a positive correlation with metabolites participating in amino acid biosynthesis and energy substrate metabolism [49]. In the HMP group, its relative abundance was the highest, likely attributable to increased protein availability in the rumen, which may enhance its role in protein degradation and amino acid synthesis. *Terrisporobacter*, known for its involvement in the in vitro anaerobic fermentation of indigestible rice straw in monogastric animals [50], exhibited the highest relative abundance in the MMP group, suggesting this group possesses greater ruminal cellulose fermentation capacity. *Ruminococcus* is a genus of organic acid-producing bacteria crucial for fiber digestion, that utilize glucose and xylose as primary fermentation products [51]. Theoretically, enhanced glucose metabolism improves the host’s uptake and utilization of nitrogen-containing compounds, implying that elevated protein levels would promote *Ruminococcus* growth and dominance. However, the present study observed the lowest *Ruminococcus* abundance in the MMP group. Lv et al. [10] reported that under low-energy diets, *Ruminococcus* abundance was lower in high-protein diets than in low-protein diets, whereas no differences were detected in high-energy diets. These findings suggested that dietary protein’s effect on *Ruminococcus* abundance is modulated by dietary energy levels. Lambs in the MMP group showed the highest abundance of the *Christensenellaceae_R-7_group*, a probiotic commonly found in both the intestinal tract and mucosal lining, which plays a role in AA metabolism [52]. Furthermore, the *Christensenellaceae_R-7_group* belongs to the *Christensenellaceae* family, a relatively newly discovered bacterial group associated with host health [52]. *Christensenellaceae* also shows a positive correlation with protein catabolism and intestinal metabolites derived from dietary animal proteins [53,54]. The *Christensenellaceae_R-7_group*, which has been demonstrated in ruminant studies to enhance rumen development and promote nutrient absorption and digestion [55,56], showed a significant positive correlation with ruminal valerate concentration in our study. Valerate is recognized as a potent histone deacetylase inhibitor and has demonstrated anti-cancer, anti-diabetic, antihypertensive, anti-inflammatory, and immunomodulatory effects in various studies [57,58]. These results indicated that the improved growth performance in MMP-fed lambs is related to the increased efficacy of probiotics in the rumen.

Microbial functional analysis revealed that “membrane transport”, “carbohydrate metabolism”, “amino acid metabolism”, “replication and repair”, “translation”, and “energy metabolism” were the most predominant predicted functions. These findings are consistent with those reported by Yang et al. [59], demonstrating that the core functions of the rumen microbiota remain relatively stable. Nevertheless, dietary MP levels were found to influence specific metabolic pathways, particularly lipid metabolism. Gebeyew et al. [60] reported that an increased abundance of *Candidatus_Saccharimonas* was associated with alterations in lipid metabolism. In the current study, the MMP group exhibited the lowest abundance of *Candidatus_Saccharimonas* along with reduced lipid metabolism, suggesting that moderate MP levels may regulate fat metabolism and decrease fatty acid absorption. Unexpectedly, the function of amino acid metabolism was not affected by the dietary MP levels. This could be attributed to the inherent characteristics of rumen microbiota, as the functional roles of non-classified bacteria may have been undervalued [60]. As noted by Ma et al. [61], PICRUSt has certain limitations in predicting microbial functions, underscoring the need for further research using metagenomic and/or metabolomic approaches to better understand the impact of MP levels on rumen functionality.

## 5. Conclusions

The moderate dietary MP level significantly improved growth performance and nitrogen utilization efficiency. This optimal MP level enhanced nitrogen retention through balanced plasma AA profiles while reducing apparent N digestibility, suggesting more efficient nitrogen utilization. The observed effects were associated with distinct rumen microbial changes, particularly an increased abundance of the *Christensenellaceae_R-7_group*, a known probiotic taxon in rumen ecosystems. Importantly, this MP level represents a balance between animal performance and environmental considerations, as it maintained growth while potentially mitigating nitrogen emissions. Based on comprehensive evaluation of physiological responses, microbial dynamics, and environmental impact, we recommend 8.66% dietary MP as the optimal level for 2–4-month-old female Hu lambs.

## Figures and Tables

**Table 1:** Ingredients and chemical composition (% of dry matter) of diets.

Item	Treatments ^1^		
	LMP	MMP	HMP
Ingredients			
Soybean meal	3.57	9.24	14.9
Corn	23.31	17.64	11.98
Bran	1.40	1.40	1.40
Bamboo leaf	22.72	22.72	22.72
Peanut seedling	40.00	40.00	40.00
Tofukasu	8.00	8.00	8.00
Premix ^2^	1.00	1.00	1.00
Chemical compositions ^3^			
Crude protein	11.9	14.2	16.0
Metabolizable protein	7.38	8.66	9.93
Neutral detergent fiber	36.1	36.4	36.6
Acid detergent fiber	26.2	26.6	27.0
Ether extract	3.77	3.68	3.58
Ca	1.36	1.38	1.39
P	0.21	0.23	0.25
Digestible energy, MJ/kg	10.3	10.3	10.3

^1^ LMP = low metabolizable protein; MMP = moderate metabolizable protein; HMP = high metabolizable protein. ^2^ Premix provided the following per kilogram of diet: vitamin A 19,000 IU, vitamin D 35,000 IU, vitamin E 900 IU, Cu 600 mg, Fe 1 g, Zn 3 g, Mn 3 g, I 50 mg, Se 5 mg, Co 40 mg, Ca 8% to 24%, NaCl 8.0 to 24.0, P 1.5% to 4.5%. ^3^ Metabolizable protein and digestible energy were predicted values and the other nutrients were measured values.

**Table 2:** Effects of dietary metabolizable protein on the growth performance of Hu lambs.

Item ^2^	Treatments ^1^			SEM	*p*-Value
	LMP	MMP	HMP		
Initial BW, kg	18.9	19.2	19.1	0.15	0.578
Final BW, kg	26.6 ^b^	28.1 ^a^	28.0 ^a^	0.27	0.008
DMI, g/d	824	823	814	10.7	0.923
ADG, g/d	155 ^b^	173 ^a^	180 ^a^	3.37	0.001
Feed efficiency ^3^, g/kg	189	211	221	6.98	0.164

^1^ LMP = low metabolizable protein; MMP = moderate metabolizable protein; HMP = high metabolizable protein. ^2^ BW = body weight; DMI = dry matter intake; ADG = average daily gain. ^3^ Feed efficiency = average daily gain/dry matter intake. ^a,b^ Means within rows with different superscript letters differ (*p* < 0.05).

**Table 3:** Effects of dietary metabolizable protein on nitrogen utilization.

Item	Treatments ^1^			SEM	*p*-Value
	LMP	MMP	HMP		
Nitrogen intake, g/d	92.0 ^c^	111 ^b^	127 ^a^	4.53	<0.001
Urinary nitrogen, g/d	1.16 ^c^	1.53 ^b^	2.75 ^a^	0.253	0.008
Fecal nitrogen, g/d	42.3	42.2	41.7	1.19	0.978
Retained nitrogen, g/d	48.6 ^c^	67.5 ^b^	82.9 ^a^	4.35	<0.001
Nitrogen apparent digestibility, %	45.9 ^a^	38.0 ^b^	32.6 ^c^	1.85	<0.001
Percent nitrogen retained, %	52.8 ^c^	60.7 ^b^	65.2 ^a^	1.78	0.002
Nitrogen retained/body weight gain, g/100 g	31.4 ^c^	39.5 ^b^	46.1 ^a^	2.01	0.001

^1^ LMP = low metabolizable protein; MMP = moderate metabolizable protein; HMP = high metabolizable protein. ^a,b,c^ Means within rows with different superscript letters differ (*p* < 0.05).

**Table 4:** Effects of dietary metabolizable protein on urinary purine derivatives and estimated metabolizable protein supply.

Item ^2^	Treatments ^1^			SEM	*p*-Value
	LMP	MMP	HMP		
Total PD, mg/d	7.41 ^b^	8.02 ^b^	10.5 ^a^	0.548	0.031
Allantoin	4.56	4.69	5.98	0.374	0.250
Uric acid	0.83 ^b^	0.94 ^b^	1.26 ^a^	0.066	0.004
Xanthine + hypoxanthine	2.01 ^b^	2.39 ^b^	3.25 ^a^	0.201	0.016
MCP ^3^, g/d	33.7 ^b^	36.4 ^b^	47.7 ^a^	2.490	0.031
IAMCP ^4^, g/d	26.9 ^b^	29.1 ^b^	38.1 ^a^	1.992	0.031
IADP ^5^, g/d	38.1 ^b^	45.0 ^b^	51.5 ^a^	1.732	<0.001
Actual MP ^6^, g/d	65.0 ^c^	74.1 ^b^	89.6 ^a^	3.353	<0.001
Metabolizability of protein ^7^, %	70.7	66.6	70.5	1.405	0.458

^1^ LMP = low metabolizable protein; MMP = moderate metabolizable protein; HMP = high metabolizable protein. ^2^ PD = purine derivative; MCP = microbial crude protein; IAMCP = intestinally absorbable microbial crude protein; IADP = intestinally absorbable dietary protein; MP = metabolizable protein; IDP = intestinal digestibility of RUP. ^3^ MCP = total purine derivatives × 70 × 6.25/(0.116 × 0.83 × 1000). ^4^ IAMCP = MCP × 0.8. ^5^ IADP = RUP × IDP; RUP and IDP were measured according to a three-step procedure. ^6^ Actual MP = IAMCP + IADP. ^7^ Metabolizability of protein = actual MP/N intake. ^a,b,c^ Means within rows with different superscript letters differ (*p* < 0.05).

**Table 5:** Effects of dietary metabolizable protein on the plasma free amino acid content of Hu lambs.

Item ^2^	Treatments ^1^			SEM	*p*-Value
	LMP	MMP	HMP		
Essential AAs, mg/L					
Arg	192 ^b^	195 ^a^	198 ^a^	0.69	0.001
His	39.9	39.9	40.1	0.27	0.968
Ile	5.00	5.01	5.12	0.034	0.178
Leu	16.4	16.6	16.9	0.10	0.132
Lys	34.9 ^b^	36.3 ^a^	36.7 ^a^	0.26	0.003
Met	19.2 ^b^	20.0 ^a^	20.4 ^a^	0.18	0.009
Phe	7.30 ^b^	7.97 ^a^	8.14 ^a^	0.110	0.002
Thr	34.0 ^b^	34.5 ^b^	36.0 ^a^	0.25	0.001
Val	22.0	23.4	23.0	0.30	0.132
Total essential AAs	371 ^b^	379 ^a^	384 ^a^	1.36	0.001
Nonessential AAs, mg/L					
Ala	9.08	9.41	9.97	0.164	0.061
Asp	44.1 ^b^	46.8 ^a^	46.4 ^a^	0.37	0.002
Cys	8.87 ^a^	8.29 ^b^	8.49 ^b^	0.071	0.001
Glu	47.6	47.8	48.1	0.20	0.574
Gly	10.1	10.3	10.5	0.09	0.254
Pro	30.5 ^b^	32.3 ^a^	32.3 ^a^	0.28	0.004
Ser	7.58	7.61	8.10	0.144	0.224
Tyr	9.30	9.24	9.26	0.033	0.808
Total nonessential AAs	167 ^b^	172 ^a^	173 ^a^	0.68	0.001

^1^ LMP = low metabolizable protein; MMP = moderate metabolizable protein; HMP = high metabolizable protein. ^2^ AA = amino acid; Arg = arginine; His = histidine; Ile = isoleucine; Leu = leucine; Lys = lysine; Met = methionine; Phe = phenylalanine; Thr = threonine; Val = valine; Ala = alanine; Asp = aspartic acid; Cys = cysteine; Glu = glutamic acid; Gly = glycine; Pro = proline; Ser = serine; Tyr = tyrosine. ^a,b^ Means within rows with different superscript letters differ (*p* < 0.05).

**Table 6:** Effects of dietary metabolizable protein on rumen fermentation parameters.

Items	Treatments ^1^			SEM	*p*-Value
	LMP	MMP	HMP		
Total VFAs ^2^, mmol/L	66.6	61.2	68.8	5.16	0.591
Acetate	46.3	41.6	47.2	3.68	0.546
Propionate	9.59	8.87	9.84	0.874	0.727
Iso-butyrate mmol/Lmmol/Lmmol/L	1.46 ^b^	1.51 ^b^	1.81 ^a^	0.061	0.007
Butyrate	6.29	6.15	6.72	0.613	0.794
Iso-valerate	2.25	2.35	2.43	0.132	0.610
Valerate	0.72	0.70	0.74	0.064	0.897
NH_3_-N ^3^, mg/L	129 ^c^	156 ^b^	170 ^a^	6.12	<0.001

^1^ LMP = low metabolizable protein; MMP = moderate metabolizable protein; HMP = high metabolizable protein. ^2^ VFAs = volatile fatty acids. ^3^ NH_3_-N = ammonia–nitrogen. ^a,b,c^ Means within rows with different superscript letters differ (*p* < 0.05).

## Data Availability

The ruminal bacterial 16S rRNA gene sequencing data were deposited in the NCBI Sequence Read Archive database (accession number: PRJNA946913).

## Supplementary Materials

The following supporting information can be downloaded at: https://www.mdpi.com/article/10.3390/ani15142147/s1, Figure S1. Rarefaction (A) and Venn diagram (B) of ruminal microbiota.

## Abbreviations

The following abbreviations are used in this manuscript:

AA	Amino acid
ADF	Acid detergent fiber
ADG	Average daily gain
ANOSIM	Analysis of similarities
AOAC	Association of Official Agricultural Chemists
BW	Body weight
CP	Crude protein
DM	Dry matter
DMI	Dry matter intake
FN	Fecal nitrogen
IADP	Intestinally absorbable dietary protein
IAMCP	Intestinally absorbable microbial crude protein
IDP	Intestinal digestibility of rumen undegraded protein
KEGG	Kyoto Encyclopedia of Genes and Genomes
LEfSe	Linear discriminant analysis effect size
MCP	Microbial crude protein
MP	Metabolizable protein
N	Nitrogen
NDF	Neutral detergent fiber
NH_3_-N	Ammonia nitrogen
NI	Nitrogen intake
OTUs	Operational taxonomic units
PCoA	Principal coordinate analysis
PD	Purine derivative
PICRUSt	Phylogenetic investigation of communities by reconstruction of unobserved states tool
QIIME	Quantitative insight into microbial ecology
RDP	Rumen degradable protein
RUP	Rumen undegradable protein
UN	Urinary nitrogen
VFA	Volatile fatty acids
