# Supplementary figures and images for: Nitrogen Utilization and Ruminal Microbiota of Hu Lambs in Response to Varying Dietary Metabolizable Protein Levels
Source: Animals (Basel). 2025 Jul 21;15(14):2147. doi: 10.3390/ani15142147 (PMC12291718; doi:10.3390/ani15142147)

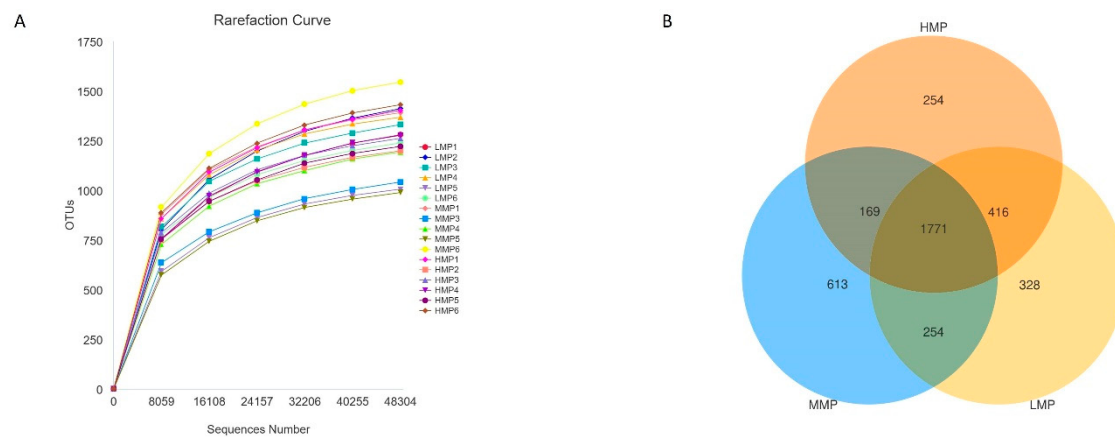

Figure S1. Rarefaction(A) and Venn diagram(B) of ruminal microbiota.

Supplement: Supplementary file 1 [file animals-15-02147-s001.zip › animals-3774876-supplementary.pdf]
